# Automatic barcode gap discovery reveals diverse clades of *Rhipicephalus* spp. and *Haemaphysalis* spp. ticks from small mammals in 'Asir, Saudi Arabia

**DOI:** 10.1186/s13071-021-05049-x

**Published:** 2021-10-19

**Authors:** Samia Q. Alghamdi, Van Lun Low, Hadil A. Alkathiry, Abdulaziz N. Alagaili, John W. McGarry, Benjamin L. Makepeace

**Affiliations:** 1 Institute of Infection, Veterinary & Ecological Sciences, University of Liverpool, 146 Brownlow Hill, Liverpool, L3 5RF UK; 2 College of Science, Al Baha University, Al Baha Province, Alaqiq, 65779-77388 Saudi Arabia; 3 Tropical Infectious Diseases Research & Education Centre, Universiti Malaya, 50603 Kuala Lumpur, Malaysia; 4 Department of Biology, Imam Muhammad Ibn Saud Islamic University, Riyadh, 13318 Saudi Arabia; 5 Department of Zoology, King Saud University Mammals Research Chair, King Saud University, Riyadh, 12372 Saudi Arabia

**Keywords:** Molecular barcoding, Ixodidae, *Meriones rex*, *Acomys dimidiatus*, Jird, Brown dog tick

## Abstract

**Background:**

The ixodid tick genera *Rhipicephalus* and *Haemaphysalis* contain several species of medical and/or veterinary importance, but their diversity in some regions of the world remains under-explored. For instance, very few modern studies have been performed on the taxonomy of these genera on the Arabian Peninsula.

**Methods:**

In this study, we trapped small mammals in the 'Asir Mountains of south-western Saudi Arabia and collected tick specimens for morphological examination and molecular barcoding, targeting three mitochondrial loci: *cox1*, 16S rRNA and 12S rRNA.

**Results:**

We obtained a total of 733 ticks (608 *Haemaphysalis* spp. and 125 *Rhipicephalus* spp.) from 75 small mammal hosts belonging to six species. All tick specimens were immature except for nine adults recovered from a hedgehog (*Paraechinus aethiopicus*). Morphologically, the *Rhipicephalus* ticks resembled *R. camicasi*, but the *Haemaphysalis* ticks showed differences in palp morphology compared with species previously described from Saudi Arabia. Phylogenetic analysis and automatic barcode gap discovery identified a novel clade of *Rhipicephalus* sp. representing most of the nymphs. This was most closely related to *R. leporis*, *R. guilhoni* and *R. linnaei*. The adult ticks and a small proportion of nymphs clustered with *R. camicasi* sequences from a previous study. Finally, the *Haemaphysalis* nymphs formed two distinct clades that were clearly separated from all reference sequences but closest to some African species.

**Conclusions:**

This apparent high level of tick diversity observed in a single study site of only ~ 170 km^2^, on a relatively small number of hosts, highlights the potential for the discovery of new tick species on the Arabian Peninsula.

**Graphical Abstract:**

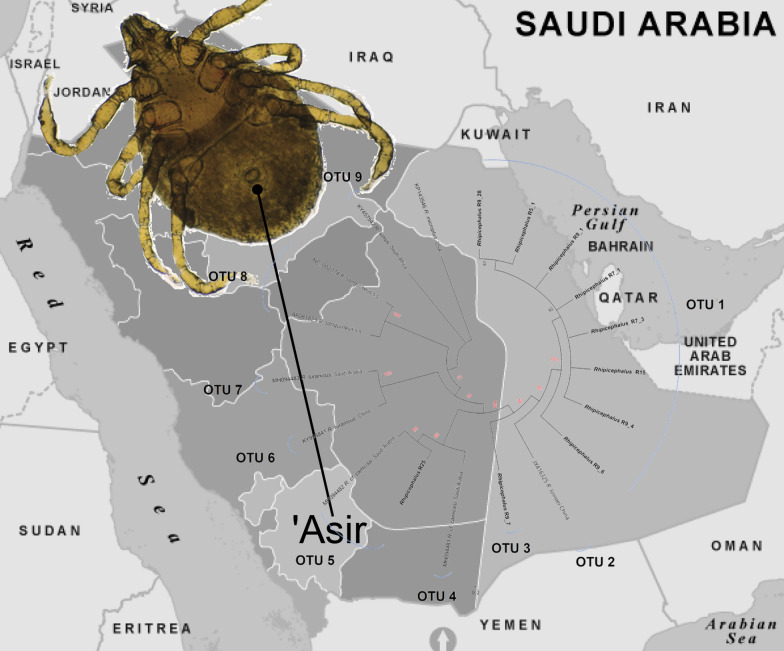

**Supplementary Information:**

The online version contains supplementary material available at 10.1186/s13071-021-05049-x.

## Background

The Ixodidae (hard ticks) is by far the most speciose family of ticks, with over 700 validly described species [1]. Until comparatively recently, our understanding of the relationships between tick species was founded almost exclusively on analysis of morphological features. Due to their large and complex genomes, whole nuclear genome data for ticks remain sparse [2] compared with insects of medical and/or veterinary importance, and investigations of possible species complexes within morphologically similar tick groups have proceeded slowly. However, molecular confirmation of tick species identity using mitochondrial barcodes and phylogenetic analyses based on concatenated mitochondrial loci, or more recently, nucleotide and amino acid datasets from whole mitogenomes, have begun to revolutionise both the taxonomic status of closely related species and the higher-level relationships between tick genera and families [3–7].

There have been increasing reports of discordance between morphological features and genetic characteristics within ixodid taxa, including *Ixodes* and *Rhipicephalus*; two of the most intensely studied genera of medical and veterinary importance. For instance, a recent study showed that certain Australian *Ixodes* spp. specimens were highly divergent genetically but morphologically indistinguishable, whereas other specimens were morphologically distinct but poorly resolved genetically [8]. Moreover, two of the most important *Rhipicephalus* spp. globally, the Asian blue tick, *R. microplus*, and the brown dog tick, *R. sanguineus*, are each now known to be formed of several distinct lineages, which are becoming recognised as distinct species [9–15]. The highly diverse genus *Haemaphysalis* has been the subject of far fewer molecular studies, although substantial discrepancies between morphology-based classification and molecular characteristics have recently been noted for this taxon too [4, 7, 16]. One generic approach to resolving species diversity using objective molecular criteria is automatic barcode gap discovery (ABGD), which is founded on the principle that the genetic divergence should be smaller within species than between species [17]. This allows a confidence limit to be assigned to intraspecific divergence, thus partitioning gene sequences into bins or operational taxonomic units (OTUs). The ABGD approach and related methods are gaining in popularity in molecular studies of ticks worldwide [18–20].

One geographic region in which the diversity of Ixodidae is under-explored is the Arabian Peninsula. A key to the ticks of Yemen was published by Hoogstraal and Kaiser [21] and for Saudi Arabia by Hoogstraal et al. [22]. Recent reports of ticks from the region have focused primarily on identification of species collected from domestic animals and pathogen screening [23–25], with a smaller number of studies on tick specimens obtained from wild hosts [26–28]. Importantly, to the best of our knowledge, no molecular data from ticks collected from wildlife in Saudi Arabia have been published to date. Here, we identify a novel clade of *Rhipicephalus* spp. ticks feeding on rodents in the 'Asir Mountains of south-western Saudi Arabia, which is molecularly distinct from sympatric specimens that cluster with *R. camicasi*. We also present preliminary evidence for two novel clades of *Haemaphysalis* spp. ticks infesting the same hosts.

## Methods

### Field site and small mammal trapping

Details of the study site and small mammal collection have been published previously [29]. Briefly, small mammals were trapped overnight in the summers of 2016 and 2017 near three villages (Al Ous', Alogl and Wosanib) on the upper escarpment of the 'Asir Mountains in south-western Saudi Arabia, between the towns of Abha and Muhayil Asir. An additional brief excursion to the same area was undertaken in October 2020. Rodents were identified morphologically with reference to the work of Harrison and Bates [30]. Molecular confirmation was performed by amplification of a cytochrome *b* gene barcode using conventional polymerase chain reaction (PCR) with primers L14841 and H15149 [31]. These rodent sequences were submitted to the Barcode of Life Data Systems (BOLD) (http://www.boldsystems.org) under project code SSS.

### Morphological examination of ticks

Mammal carcasses were examined for ticks with the naked eye and then under a dissecting microscope. Ticks were removed with fine forceps, fixed in 70% ethanol and maintained at 4 °C prior to enumeration. Approximately 5% of specimens from each host were selected for morphological or molecular analysis, prioritising nymphs over larvae due to the low DNA yields and problems in identification associated with the latter. Semi-engorged immature stages selected for morphological examination were placed in distilled water for 10 min, transferred to a macerating solution (10% potassium hydroxide) and incubated at 37 °C for up to 10 min until the cuticle had cleared sufficiently to visualise key morphological features. The specimens were again placed in distilled water for 10 min and then dehydrated serially using 50%, 70% and 100% ethanol (10 min at each concentration). Finally, specimens were transferred to a glass slide with a drop of DPX mountant (VWR International), covered and examined using an Axio Imager M2 microscope with ZEN 2011 imaging software (Zeiss). Adult ticks (males only in this study, as females were fully engorged) were examined directly from 70% ethanol under a dissecting microscope without further processing. Morphological features of the ticks were compared with those described in keys and other taxonomic reference works for ticks, focusing on the Middle East, Southern Europe and North Africa [14, 21, 22, 32–38].

### DNA extraction, PCR and sequencing

DNA extraction was performed with a DNeasy Blood & Tissue Kit (Qiagen) according to the manufacturer’s instructions. In the case of immature ticks, DNA was extracted from the whole specimen, whereas for adults, DNA extraction was performed on the anterior portion only to reduce carryover of the blood meal in engorged specimens. The amplification of fragments of three mitochondrial loci (*cox1*, 12S rRNA and 16S rRNA) was attempted for each specimen (Table 1) using previously published primers from Low and Prakash [10], Beati and Keirans [39], and Black and Piesman [6], respectively. Expected product sizes were 550 bp for *cox1*, 336 bp for 12S rRNA and 460 bp for 16S rRNA. The PCR assays were performed on a T1 Thermoblock thermocycler (Biometra) using BioMix Red reaction mix (Meridian Bioscience) in 20-μl volumes containing 5 μl DNA template. Following agarose gel electrophoresis, PCR products were purified using a QIAquick PCR Purification Kit (Qiagen) and sequenced in both directions by Eurofins Genomics.

**Table 1:** Tick specimens examined in this study, origin and sequences obtained

Tick sample ID	Tick genus	Host species	Location	Year	Habitat type	Loci sequenced^a^		
						*cox1*	16S	12S
R11_1	*Rhipicephalus*	*M. rex*	Alogl	2017	Agricultural	Y	Y	N
R11_2						Y	N	N
R11_3						Y	N	N
R12		*M. rex*	Alogl	2017	Agricultural	N	Y	N
R13		*A. dimidiatus*	Alogl	2017	Agricultural	Y	N	Y
R15		*M. rex*	Alogl	2017	Agricultural	Y	Y	Y
R22		*M. yemeni*	Alogl	2017	Montane	Y	N	N
R25		*A. dimidiatus*	Wosanib	2017	Montane	Y	Y	Y
R29^b^		*M. rex*	Wosanib	2017	Agricultural	N	Y	Y
R3		*M. rex*	Alogl	2017	Agricultural	Y	N	N
R30		*M. rex*	Wosanib	2017	Agricultural	Y	N	N
R39		*A. dimidiatus*	Wosanib	2017	Montane	N	Y	N
R4		*M. rex*	Alogl	2017	Agricultural	N	Y	N
R46_3		*A. dimidiatus*	Wosanib	2017	Montane	N	Y	N
R5_1		*M. rex*	Alogl	2017	Agricultural	Y	Y	Y
R5_2						Y	N	N
R5_3						Y	N	N
R5_4						Y	N	N
R7_1		*M. rex*	Alogl	2017	Agricultural	Y	Y	Y
R7_2						Y	N	N
R7_3						Y	Y	Y
R7_4						Y	N	N
R8		*M. rex*	Alogl	2017	Agricultural	Y	Y	N
R9_1		*M. rex*	Alogl	2017	Agricultural	Y	Y	Y
R9_2						N	Y	N
R9_28						Y	Y	Y
R9_3						Y	Y	N
R9_4						Y	Y	Y
R9_5						N	Y	Y
R9_6						Y	Y	Y
R9_7						Y	Y	Y
R9_8						Y	N	Y
H1_1		*P. aethiopicus*	Wosanib	2020	Montane	N	Y	Y^c^
H1_2						Y	Y	Y^c^
R3a_1	*Haemaphysalis*	*A. dimidiatus*	Al Ous'	2016	Montane	NA	Y	NA
R3a_2						NA	Y	NA
R3a_3						NA	Y	NA
R3a_7						NA	Y	NA
R6a	*Haemaphysalis*	*A. dimidiatus*	Al Ous'	2016	Montane	NA	Y	NA
R20a	*Haemaphysalis*	*A. dimidiatus*	Al Ous'	2016	Montane	NA	Y	NA
R44_1	*Haemaphysalis*	*M. musculus*	Alogl	2017	Montane	NA	Y	NA
R44_2						NA	Y	NA

^a^Y, sequence obtained; N, sequence not obtained; NA, sequence amplification not attempted

^b^All sequences were obtained from individual nymphs except for R29, which was a pool of six larvae

^c^Sequences obtained were too short (~ 200 bases) to include in phylogenetic analyses

### Phylogenetic analysis and automatic barcode gap discovery

Reference sequences were selected based on Low and Prakash [10], Chandra et al. [25] and Kanduma et al. [20] for *Rhipicephalus* spp., and Hornok et al. [7] for *Haemaphysalis* spp. Additional closest reference nucleotide sequences displayed in the Basic Local Sequence Alignment Tool [40] were also included for phylogenetic tree construction. All sequences were preliminarily aligned using CLUSTAL X [41] and edited using BioEdit [42]. Phylogenetic relationships were inferred using the neighbour-joining method in MEGA X [43]. The neighbour-joining bootstrap values were estimated using 1000 replicates with Kimura’s two-parameter model of substitution (K2P distance), and bootstrap proportions of > 70% were considered well supported [44]. Gaps and missing data were eliminated. Statistical congruence was calculated using a partition homogeneity test implemented in PAUP 4.0b10 [45]. No significant differences were found among separate gene regions (*P* = 0.800); hence, *cox1*, 12S and 16S sequences were concatenated for further analyses. To assess the genetic divergence of taxa, uncorrected (p) pairwise genetic distances among species were estimated using PAUP 4.0b10 [45]. The species boundary among tick taxa was assigned by automatic barcode gap discovery (ABGD) analysis performed on the web server using the Kimura (K80) TS/TV model. Entity recognition was based on the suggested partition at *P* = 0.01 [17].

## Results

### Material obtained and examined

We obtained 75 small mammal hosts across the three sites, which belonged to six species (Table 2): the eastern spiny mouse (*Acomys dimidiatus*), king jird (*Meriones rex*), Yemeni mouse (*Myomyscus yemeni*), black rat (*Rattus rattus*), house mouse (*Mus musculus*) and desert hedgehog (*Paraechinus aethiopicus*). These were infested with a total of 733 ticks (608 *Haemaphysalis* spp. and 125 *Rhipicephalus* spp.), all of which were immature except for nine adults (seven males and two females) recovered from the hedgehog. The overall prevalence of tick infestation was 70.7%, with a mean abundance per host of 9.8. Most subsampled specimens (80–95%) from each host were prioritised for molecular analysis, including pathogen screening (to be reported separately), and we focused primarily on *Rhipicephalus* spp. due to its greater potential regional importance as a disease vector. All specimens subjected to PCR [*Rhipicephalus* spp. nymphs (*n* = 33), one pool of *Rhipicephalus* spp. larvae, and eight *Haemaphysalis* spp. nymphs] generated at least one mitochondrial gene sequence (Table 1). At least two specimens per life cycle stage of each tick genus were examined morphologically.

**Table 2:** The number of host species trapped by location

Village	GPS coordinates	Host species (*n*)					
		*A. dimidiatus*	*M. rex*	*M. musculus*	*M. yemeni*	*R. rattus*	*P. aethiopicus*
Al Ous'	18.27641, 42.320611	33	0	0	0	1	0
Wosanib	18.315641, 42.211478	10	5	0	2	0	1
Alogl	18.34654, 42.31654	2	13	3	6	0	0

### Morphological features

**Fig. 1:**
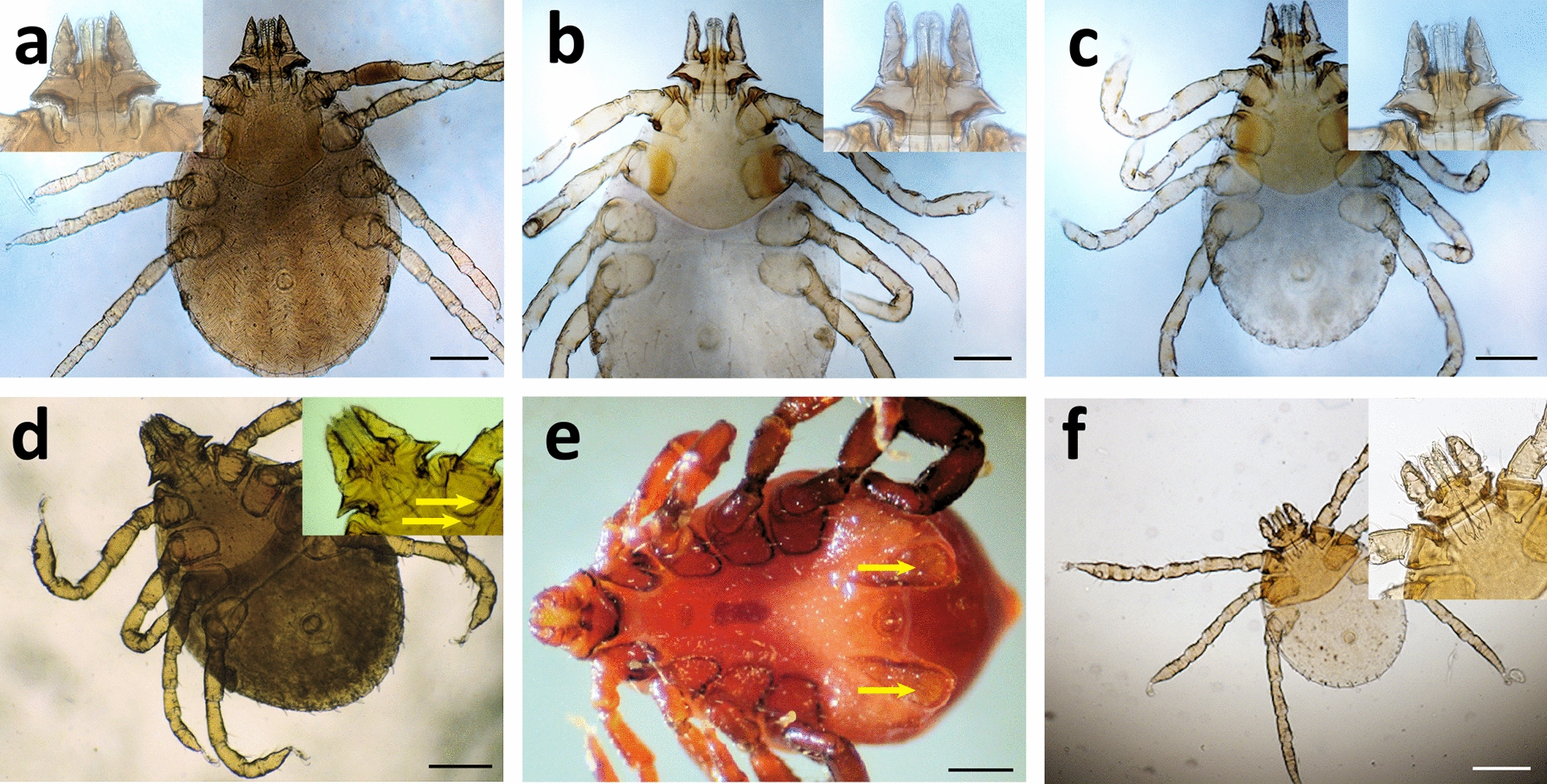
Morphology of *Rhipicephalus* spp. ticks from 'Asir. **a–c** Nymphs from Alogl (**a**, **c**) and Al Ous' (**b**) displaying variation in the shape of the palps (insets) and extent of the dorsal shield. **d** Nymph from Wosanib. Inset shows poorly defined spurs (arrows) on coxa I. **e** Adult male from Wosanib. Note the shape of adanal plates (arrows). **f** Larva from Wosanib. Inset displays details of the gnathostome. All scale bars 200 μm; except in **e**, 500 μm

*Rhipicephalus* spp. nymphs displayed variation in the length and shape of the palps as well as the appearance of the scutum, which slightly overlapped coxa III in some individuals only (Fig. 1c, d). Nymphs exhibited a highly reduced external spur on coxa I, and the internal spur appeared vestigial (Fig. 1d). According to the works of Pegram et al. [35, 36] on the *R. sanguineus* group, these features of the spurs together with the ratio of length to width of the capitulum would position these specimens closer in morphology to *R. camicasi* than to *R. turanicus* or *R. sanguineus* sensu lato (s.l.). In addition, the adanal plates of the adult males (Fig. 1e) lacked the distinctly concave shape proximal to the anus reported by Nava et al. [14] in their re-description of *R. sanguineus* sensu stricto (s.s.).

**Fig. 2:**
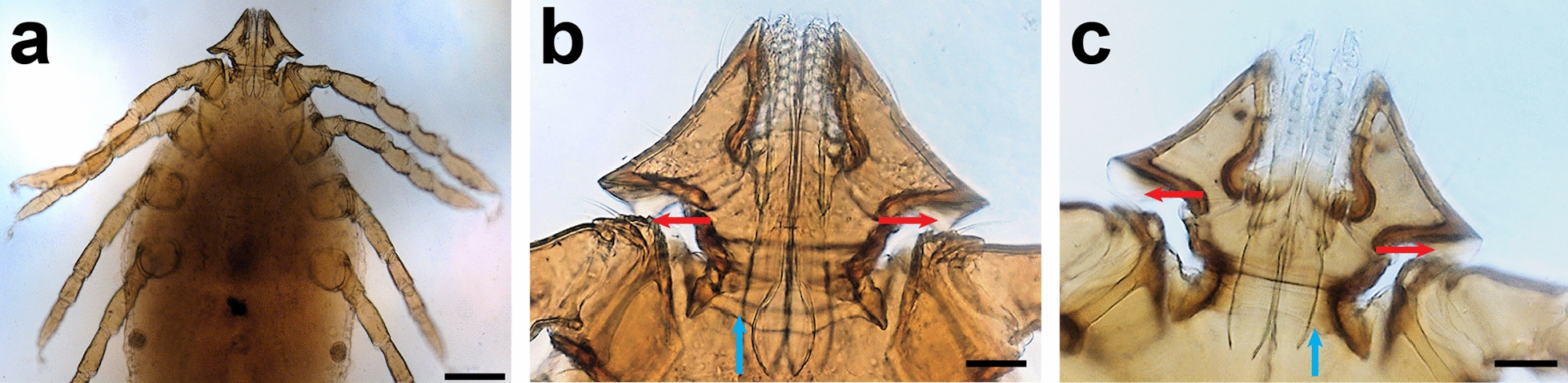
Morphology of *Haemaphysalis* nymphs from Al Ous'. **a** Overview of a specimen displaying the posteriorly flared palps. **b** Detail of the gnathostome from **a**. Note the triangular spurs on palp segment I (red arrows) and convex posterior margin to basis capitulum (blue arrow). **c** A different specimen displaying spurs on palps (red arrows) and straight posterior margin to basis capitulum (blue arrow). Scale bars, 200 μm (**a**); 50 μm (**b**, **c**)

The *Haemaphysalis* spp. nymphs displayed palps that were flared posteriorly (Fig. 2), which according to Hoogstraal et al. [22] is a feature of *H. erinacei* that distinguishes it from *H. sulcata*. However, the ventral spur on palp segment I (Fig. 2b) had a triangular profile unlike that of *H. erinacei*. Since Hoogstraal and Kaiser [21] and Hoogstraal et al. [22] also reported *H. leachi* from the Arabian Peninsula, we consulted the descriptions and re-descriptions of this species and the closely related *H. elliptica* from Africa [32, 37]. The posterior margin of the basis capituli in both of these species is convex, but in some of the specimens from 'Asir, it is straight (compare Fig. 2b, c).

### Sequence analysis of *Rhipicephalus* spp.

**Fig. 3:**
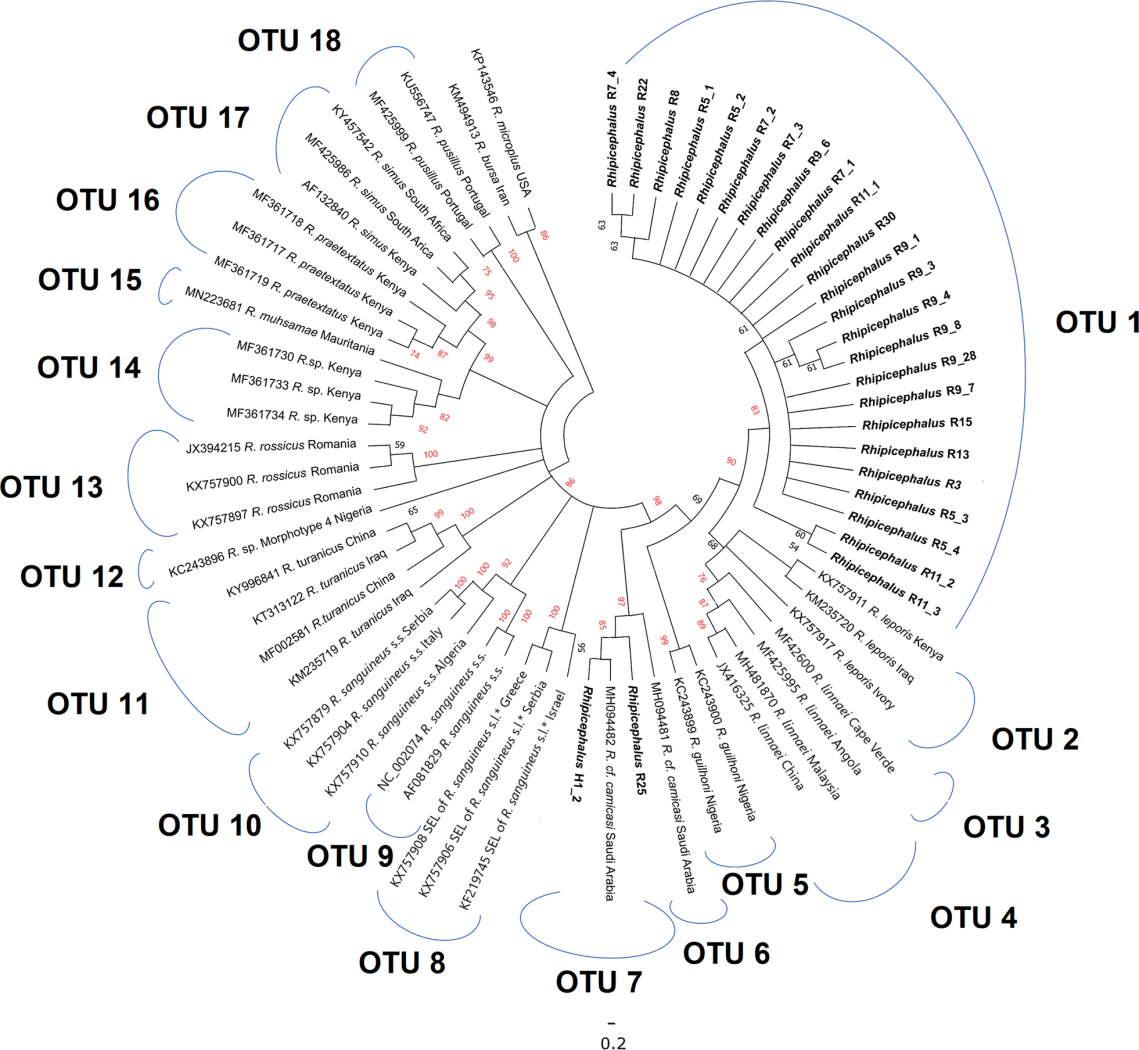
Neighbour-joining phylogenetic tree of *Rhipicephalus* taxa based on 254 bp of *cox1* sequences. Bootstrap values are shown on the branches. Sequences generated from the present study are indicated in bold type. Well-supported branches (> 70% bootstrap proportion) are indicated by red labels. *SEL = south-eastern European lineage

At least one mitochondrial gene sequence was amplified and sequenced successfully from a total of 33 *Rhipicephalus* spp. adult or nymphal tick specimens and one pool of larvae, obtained from two villages and four species of small mammal host (Table 1). The *Rhipicephalus* spp. phylogeny based on *cox1* indicated that the vast majority of nymphal specimens belonged to a single, novel clade with 90% bootstrap support; this was distinct from all other *Rhipicephalus* spp. included in the analysis (Fig. 3). The novel clade exhibited closest relationships with *R. leporis*, *R. guilhoni* and *R. linnaei*. In contrast, a single nymph (R25 from host *A. dimidiatus* in Wosanib) clustered with an adult specimen from the current study (H1_2 from host *P. aethiopicus*, also from Wosanib) and previously published sequences from “*R*. cf. *camicasi*” from Riyadh Province. The novel lineage was separated from other species by a minimum genetic distance of 2.2% (for *R. leporis*) to a maximum of 15.4% (for *R. simus*) (Additional file 1: Table S1). The ABGD analysis delimited 18 operational taxonomic units (OTUs) and supported the novel clade comprising most nymph specimens (OTU 1) as a distinct taxon (Fig. 3).

**Fig. 4:**
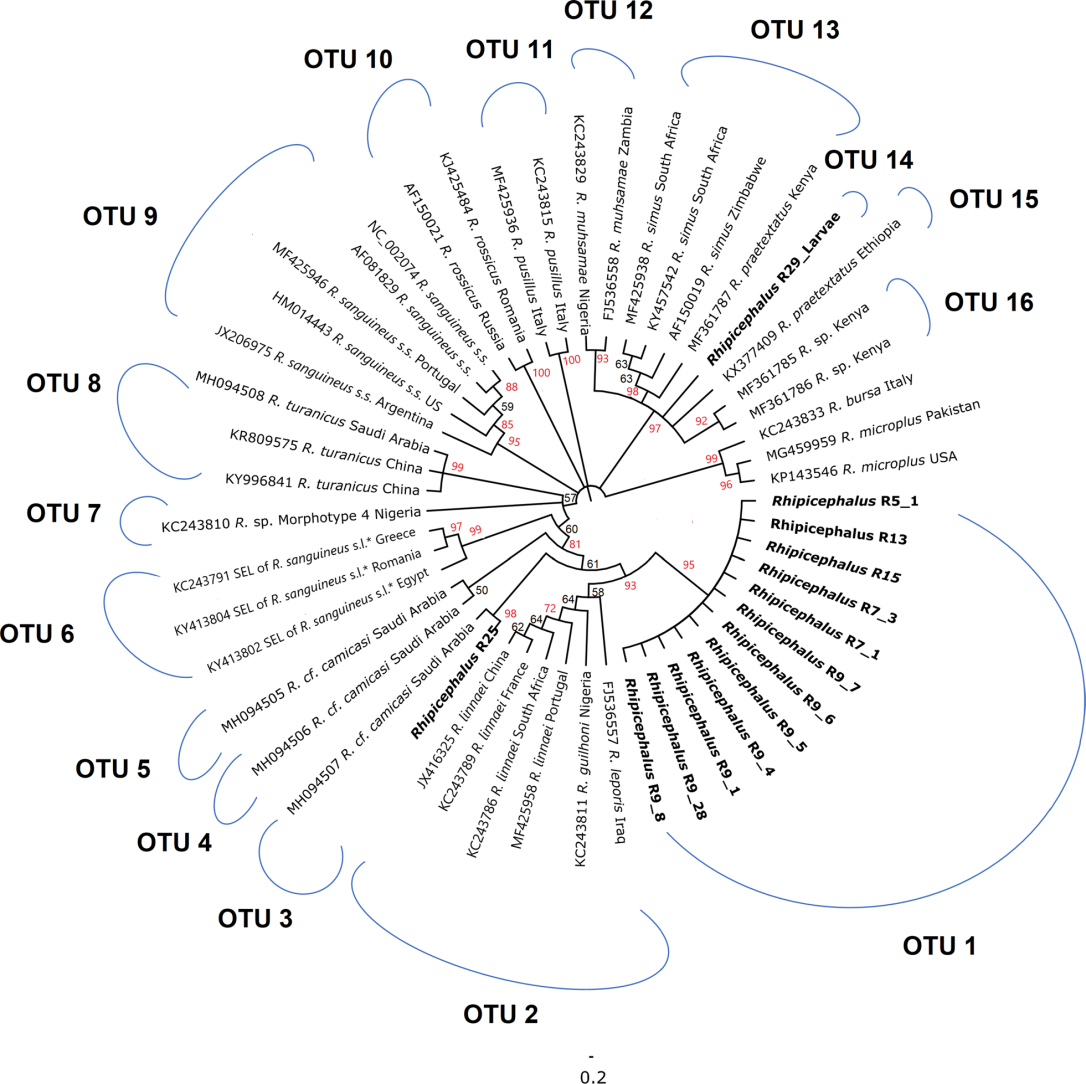
Neighbour-joining phylogenetic tree of *Rhipicephalus* taxa based on 222 bp of 12S rRNA sequences. Bootstrap values are shown on the branches. Sequences generated from the present study are indicated in bold type. Well-supported branches (> 70% bootstrap proportion) are indicated by red labels. *SEL = south-eastern European lineage

For 12S rRNA, the novel lineage was also resolved for all nymphs except R25 (> 90% bootstrap support). The clade differed from other members of the genus with lower genetic distances of 1.8% (for *R. leporis*) to 11.1% for the *R. simus* complex (including an unidentified *Rhipicephalus* sp. from Kenya; Additional file 1: Table S2). The ABGD analysis identified 16 OTUs, and although lower interspecific genetic distances were observed, the delimitation analysis demonstrated the novel lineage as a distinct OTU (Fig. 4). The “*R.* cf. *camicasi*” specimens from the previous study in Riyadh Province (obtained from camels and a dog) were split into three distinct OTUs, suggesting cryptic diversity in this species. One of these (from a camel) clustered with nymph specimen R25. Interestingly, the pool of six larvae (R29 from Wosanib) was placed in a unique OTU separated from all nymph specimens (Fig. 4). This was most closely related to members of the *R. simus* complex from Africa, especially *R. praetextatus*; indeed, the larval pool was not differentiated from the *R. simus* complex in the PAUP analysis (Additional file 1: Table S2).

**Fig. 5:**
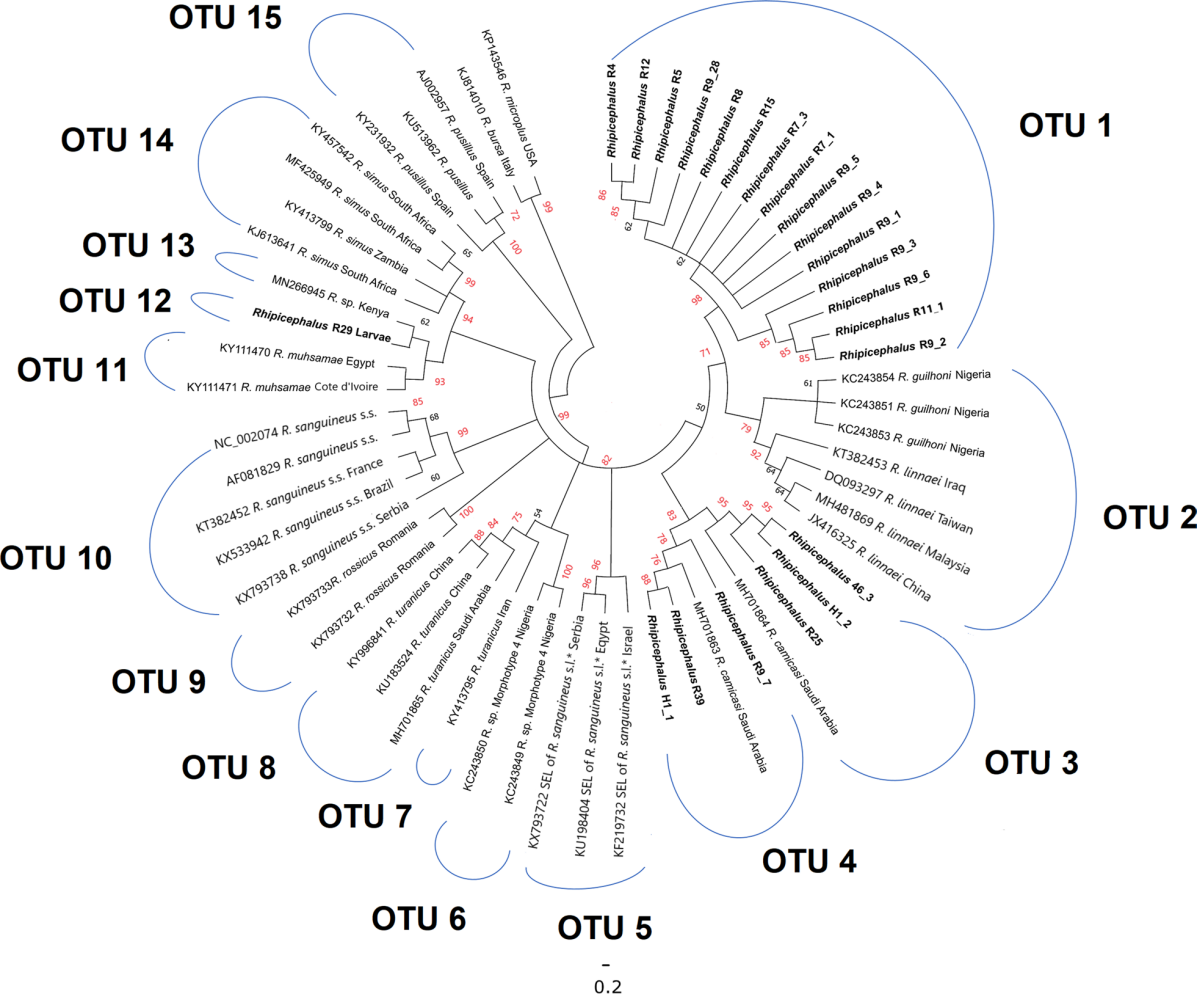
Neighbour-joining phylogenetic tree of *Rhipicephalus* taxa based on 236 bp of 16S rRNA sequences. Bootstrap values are shown on the branches. Sequences generated from the present study are indicated in bold type. Well-supported branches (> 70% bootstrap proportion) are indicated by red labels. *SEL = south-eastern European lineage

In the case of 16S rRNA, the novel lineage (> 70% bootstrap support) was also distantly separated from other members of the genus with genetic distances ranging from 4.1% (for *R. guilhoni*) to 12.3% (for *R. muhsamae*) (Additional file 1: Table S3). A total of 15 OTUs were delimited, one of which was associated with the novel lineage (Fig. 5). *Rhipicephalus* cf. *camicasi* comprised two OTUs, populated by adult specimens from *P. aethiopicus*, four nymph specimens and the previously published sequences from specimens collected from camels in Riyadh Province. An incongruence was noted for one of the tick samples, nymph R9_7 from Alogl, which was classified in the novel lineage by *cox1* and 12S rRNA genes but clustered with *R.* cf. *camicasi* OTU 4 by 16S rRNA (Fig. 5). The pool of larvae (R29) formed its own OTU (#12 in Fig. 5) that was most closely related to a sequence (OTU 13) from an unidentified *Rhipicephalus* sp. collected from a dog in Kenya (GenBank: MN266945).

**Fig. 6:**
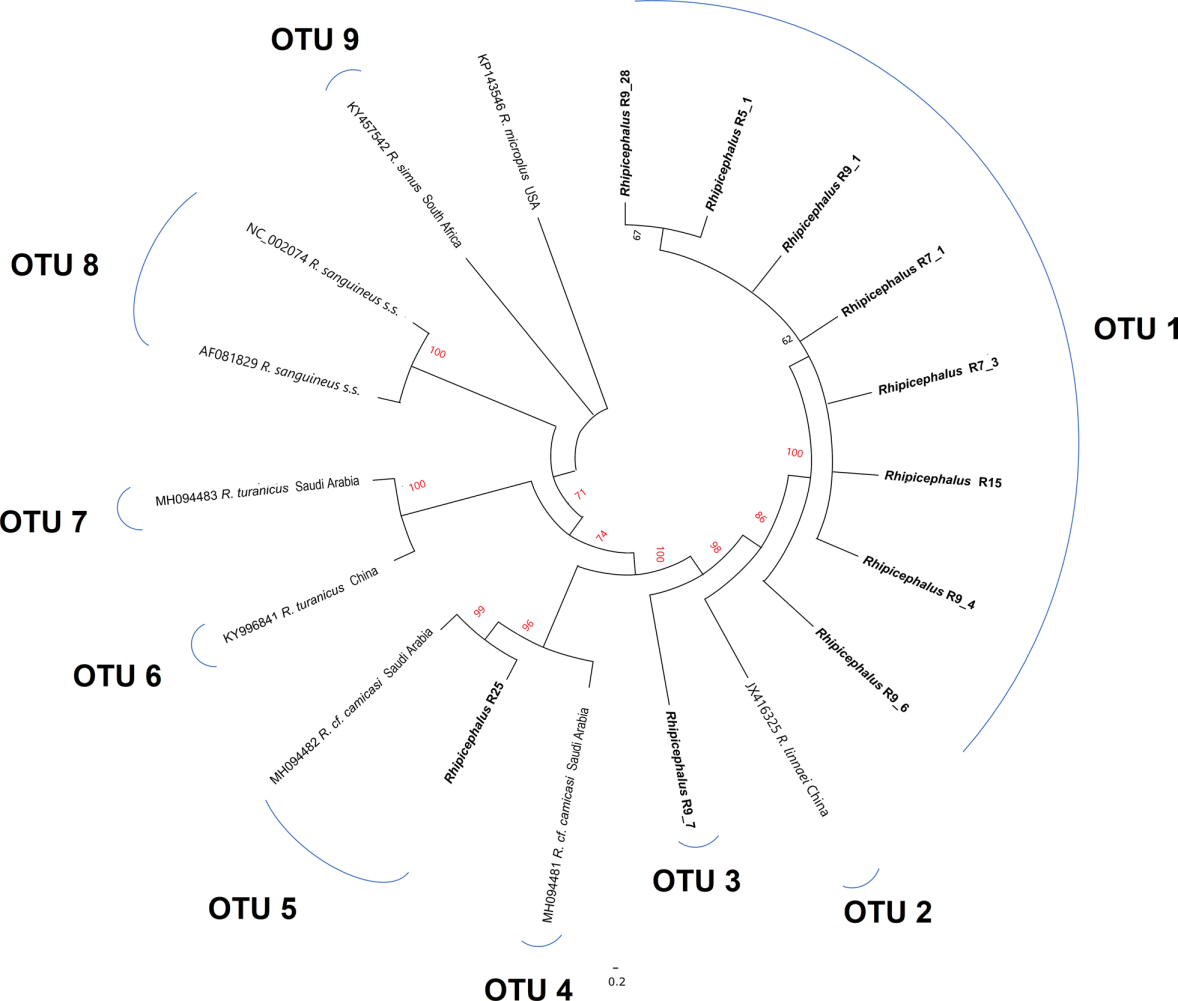
Neighbour-joining phylogenetic tree of *Rhipicephalus* taxa based on 716 bp of concatenated *cox1* + 12S rRNA + 16S rRNA sequences. Bootstrap values are shown on the branches. Sequences generated from the present study are indicated in bold type. Well-supported branches (> 70% bootstrap proportion) are indicated by red labels

Sufficient sequence data were obtained from 10 nymph specimens for a concatenated analysis of *cox1*, 12S rRNA and 16S rRNA genes alongside references for *R. sanguineus* s.s., *R. linnaei*, *R*. cf. *camicasi*, *R. turanicus* and *R. simus*. The novel clade comprised eight specimens and was distinct from all references (bootstrap support 86%), demonstrating closest affinity with *R. linnaei* (Fig. 6). In concordance with the single-gene trees, specimen R25 clustered with one of two *R*. cf. *camicasi* OTUs, whereas the incongruent specimen R9_7 formed its own OTU in proximity to *R. linnaei* (Fig. 6). As only short sequences (~ 200 bases) for 12S rRNA could be obtained from the two adult ticks from *P. aethiopicus,* they were excluded in the concatenated analysis. However, these short sequences exhibited 100% identity with the previously published *R*. cf. *camicasi* sequences from Riyadh Province (GenBank MH094506 and MH094507 from camel hosts).

### Sequence analysis of *Haemaphysalis* spp.

**Fig. 7:**
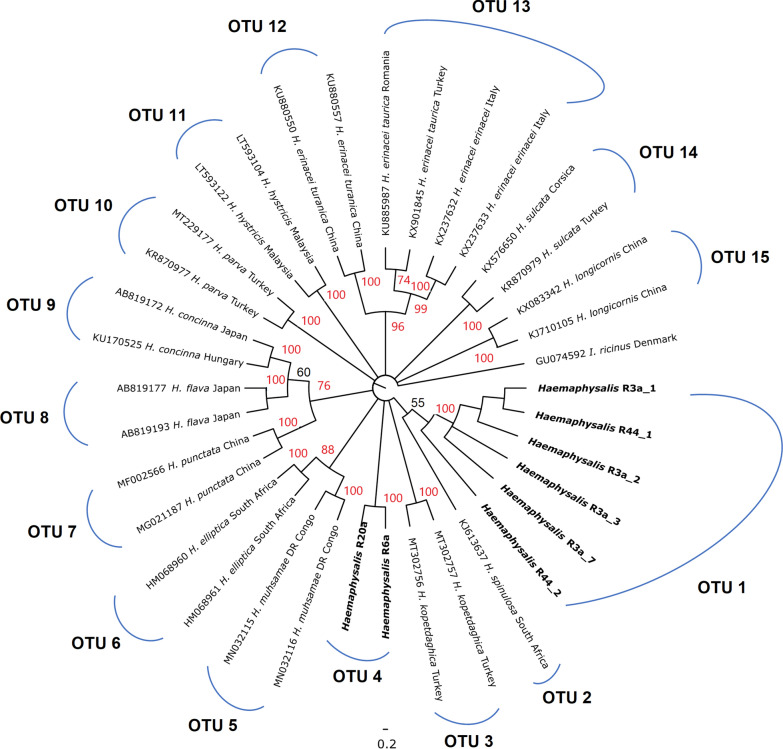
Neighbour-joining phylogenetic tree of *Haemaphysalis* taxa based on 329 bp of 16S rRNA sequences. Bootstrap values are shown on the branches. Sequences generated from the present study are indicated in bold type. Well-supported branches (> 70% bootstrap proportion) are indicated by red labels

The *Haemaphysalis* nymph samples collected in this study were resolved robustly into two lineages (100% bootstrap support) in the 16S rRNA phylogenetic tree (Fig. 7). While OTU 1 demonstrated a sister relationship with *H. spinulosa* from South Africa (genetic distance, 7.4%), OTU 4 showed closer relationships with *H. muhsamae* and *H. elliptica,* also from sub-Saharan Africa, with genetic distances of 6.8% and 8.2%, respectively (Additional file 1: Table S4). The species delimitation analysis split the Saudi specimens and references into a total of 15 OTUs, with the Saudi nymphs distinctly separated from all other species included in the analysis (Fig. 7). Notably, these two novel OTUs did not segregate by geographic location (Table 1), with OTU 1 containing specimens from both Alogl (*M. musculus* as host) and Al Ous' (*A. dimidiatus* as hosts).

## Discussion

In this study, we found widespread tick infestations represented by two genera feeding on small mammals in a relatively small region (approximately 170 km^2^) in the 'Asir Mountains. The tick abundance per host (9.8) was moderately high compared with previous studies of ticks on rodents in Saudi Arabia; for instance, in Riyadh Province, the mean abundance on gerbils was < 1, and in Ta'if (Makkah Province) it was 0.6–6.2 on gerbils and 1.4 on *A. dimidiatus* [27]. However, in Ha'il Province in the north, a mean tick abundance of ~ 20 was recorded on *R. rattus* in the most heavily infested geographical sites, although the average abundance on *A. dimidiatus* was only 3.2 [28]. Remarkably, the *Rhipicephalus* and *Haemaphysalis* ticks recovered from 'Asir not only were genetically diverse, comprising four and two OTUs, respectively, but all but one (*R. camicasi*) of these OTUs appeared to be novel. The strongest evidence for a previously unrecognised taxon was for *Rhipicephalus* OTU 1, which formed a distinct clade in the *cox1*, 12S rRNA, 16S rRNA and concatenated analyses. This clade was found on three species of rodent hosts trapped in agricultural areas surrounding the villages of Alogl and Wosanib. It was most closely related to *R. leporis*, *R. guilhoni* and the “tropical lineage” of *R. sanguineus* s.l. (recently identified as *R. linnaei* [46]). Due to the limited number of sequenced mitochondrial markers available for *R. leporis* and *R. guilhoni*, we were only able to include *R. linnaei* and more distantly related *Rhipicephalus* spp. in the concatenated phylogeny, but this analysis clearly separated the novel OTU 1 from *R. linnaei*.

Prior phylogenetic analyses have sometimes assigned *R. leporis* and *R. guilhoni* to the same clade as *R. linnaei*, along with *R. camicasi*, depending on the loci included [14, 15, 47–49]. The taxonomy and biogeography of the *R. sanguineus* group are notoriously complex due to their morphological similarity and the tendency for different species or clades to be spread worldwide on domestic hosts. Estrada-Peña et al. [38] consider *R. guilhoni* and *R. camicasi* as tropical species that have invaded Palearctic regions, whereas *R. leporis* appears to be a Palearctic species that has been introduced into sub-Saharan Africa [47]. There are few molecular data available for *R. camicasi*, but the sequences provided by Chandra et al. [25] for “*R*. cf. *camicasi*” from Riyadh Province are clearly distinct from available references for other *Rhipicephalus* spp. and clustered with a small proportion of our nymph specimens from rodents. To add further to the complexity, *R. camicasi* from Saudi Arabia did not form a single OTU in our analyses, including in the concatenated phylogeny.

*Rhipicephalus camicasi* was originally described from Northeast Africa in 1976 [33]. It was not included in the tick fauna of Saudi Arabia by Hoogstraal et al. [22], who listed only two native *Rhipicephalus* spp. (*R. sanguineus* s.l. and *R. turanicus*), excluding the subgenus *Boophilus*. However, they noted the presence of unidentified *Rhipicephalus* spp. on numerous mammalian hosts, including *A. dimidiatus*, *M. rex* and *M. musculus*. Subsequently, Pegram et al. [36] stated that *R. camicasi* could be found on livestock (ruminants, camels and donkeys) in Yemen and Saudi Arabia without details of specific locations. More recently, *R. camicasi* has been reported from sheep in Makkah Province [50] and from camels and dogs in Riyadh Province [25, 26], as well as from *A. dimidiatus* (as nymphs and larvae) in Ta'if [27]. To the best of our knowledge, *R. camicasi* has not been reported from a hedgehog host previously worldwide [38]. Our incidental finding of *R. camicasi* on a single *P. aethiopicus* in this study should be followed by a targeted survey to determine whether this common and widespread host acts as a vehicle or reservoir to maintain *R. camicasi* populations nationwide.

Very few studies have attempted to identify ticks from small mammal hosts from Saudi Arabia or Yemen previously. However, the classic wild mammal survey of Yemen (which borders 'Asir) by Sanborn and Hoogstraal [51] reported *R. simus*, *R. sanguineus* s.l. and *Ornithodoros* sp. from *M. musculus*; *H. leachi* and *R. simus* from *A. dimidiatus*; and *R. simus* and “*Ixodes* sp. nov.” from *M. rex*, among a wide range of other hosts examined. Similar host–ectoparasite relationships were recorded by Hoogstraal et al. [22] for Saudi Arabia, with the addition of immature *Hyalomma* spp. observed on all three rodent species. Our finding of *Rhipicephalus* larvae on *M. rex* that appeared to be closely related to the *R. simus* complex supports these early observations of Hoogstraal regarding the introduction of African *Rhipicephalus* spp. into the Arabian Peninsula. Asiry and Fetoh [28] described *R. turanicus* infestations on *A. dimidiatus*, alongside *R. sanguineus* s.l. and *R. turanicus* feeding on *R. rattus*, from Ha'il Province. Notably, the most recent prior survey by Harrison et al. [27] echoed the work of Hoogstraal et al. [22] in reporting the presence of an unidentified immature *Rhipicephalus* sp. on rodents in Riyadh and Ta'if. It was most common on *M. rex* in Ta'if, but was also found on *Meriones lybicus* in Riyadh and in smaller numbers on *Gerbillus nanus* in both locations. Only a single specimen was found on *A. dimidiatus* (in Ta'if), a host species on which it was apparently outcompeted by *R. camicasi* (see above). However, no morphological description (in particular, how the specimens were differentiated from *R. camicasi*) or molecular barcode was provided for this unidentified *Rhipicephalus* sp. Overall, these studies from Arabia highlight distinct differences compared with the wider Middle East, as a recent systematic review reported that *Hyalomma rhipicephaloides* and *Ixodes eldaricus* were the most prevalent ticks found on rodents in the whole region, representing 69.7% and 15.7% of ticks identified, respectively [52].

The only native *Haemaphysalis* spp. recorded from Saudi Arabia in Hoogstraal et al. [22] were *H. erinacei* and *H. sulcata*; while in Yemen, *H. leachi* (presumably introduced from Africa) was reported on *A. dimidiatus* [21, 51]. Prior to the emergence of severe fever with thrombocytopenia syndrome virus and the global spread of its vector, *H. longicornis*, molecular analyses of the genus *Haemaphysalis* had been relatively limited [53]. However, sufficient data are available to conclude that neither the morphology nor the 16S rRNA sequences of our *Haemaphysalis* spp. specimens are fully compatible with species previously recorded from Arabia. The two distinct OTUs we identified exhibited closest relationships with African *Haemaphysalis* spp. (*H. spinulosa*, *H. muhsamae* and *H. elliptica*) that primarily parasitise carnivores or erinaceids in the adult stage and rodents as immature stages [37, 54]. The previous surveys of rodent ticks conducted in Arabia (see above) suggest that *Haemaphysalis* spp. are restricted (or at least more abundant) in Yemen and southern Saudi Arabia compared with more northern regions. Whether the novel *Haemaphysalis* OTUs represent undescribed species native to the southern Arabian Peninsula will require further investigations, including locating adult specimens for comprehensive morphological and molecular analyses.

This first molecular analysis of ticks collected from rodents in the Arabian Peninsula raises many questions about the evolution and distribution of *Rhipicephalus* spp. and *Haemaphysalis* spp. in this understudied region. For instance, the taxonomic status and native geographical range of *R. camicasi* is still poorly defined, especially with respect to its relationship with *R. linnaei*. As highlighted by Hekimoglu et al. [55], Asia Minor and the Middle East constitute a bridge between Europe and Africa in the evolutionary history of the *R. sanguineus* group, in which the role of *R. camicasi* remains enigmatic. A limitation of our study was that in order to maximise DNA yields for multiple PCR assays, a portion of each specimen was not retained as a voucher [56] prior to DNA extraction. Hence, it is not clear whether *R. camicasi* and *Rhipicephalus* OTU 1 are morphologically distinct in the immature stages, which would be suggested by the work of Harrison et al. [27]—if OTU 1 is indeed the species they recorded from Riyadh and Ta'if.

The strongest evidence that OTU 1 constitutes a distinct species is that the ABGD analysis consistently binned known *Rhipicephalus* spp. at each locus into separate OTUs, while OTU 1 was delimited with moderate to strong phylogenetic support (bootstrap values ≥ 70%; [44]) for each locus but especially in the concatenated tree. However, the genetic distance of OTU 1 from *R. leporis* was modest (~ 2%) at the *cox1* and 12S rRNA loci; while as noted above, a lack of 16S rRNA data for this species prevented its inclusion in the concatenated analysis. In a previous tick barcoding study across multiple genera using neighbour-joining analysis only, optimal species delimitation boundaries were considered to be 5.3% for 16S rRNA and 6.1% for *cox1*, although few *Rhipicephalus* spp. were included in this work [18]. The formal characterisation of *Rhipicephalus* OTU 1 will require sampling of adult stages from the environment [or host(s)—which of course remain unknown currently], followed by detailed morphological and molecular comparisons with closely related species and (ideally) laboratory experiments to determine reproductive compatibility. This would provide substantive evidence that OTU 1 is a novel species or a subspecies, rather than just a divergent mitochondrial haplotype. Indeed, although mitochondrial versus nuclear marker-based phylogenies for ticks are generally congruent [3, 20], nuclear–mitochondrial discordance has been observed within tick species previously [57]. Moreover, the incongruent results between mitochondrial loci for specimen R9_7 could indicate hybridisation between *Rhipicephalus* OTU 1 and *R. camicasi*.

## Conclusions

In a small region of the 'Asir Mountains in south-western Saudi Arabia, small mammals were found to be infested with *Rhipicephalus* spp. and *Haemaphysalis* spp. ticks that formed four and two clades, respectively, by the ABGD method. In addition to two clades of *R. camicasi*-like adult and nymphal ticks and one clade of *R. simus*-like larvae, a novel OTU composed of *Rhipicephalus* nymphs was found infesting three species of rodent hosts. It was related to, but distinct from, *R. leporis*, *R. guilhoni* and *R. linnaei*. Taken together, our findings indicate that a hotspot of tick diversity may exist in the 'Asir Mountains that deserves further faunistic, ecological and genetic investigations. This conclusion is supported by the fact that most previous studies of tick genetic diversity have identified population structuring at the level of whole countries or continents rather than at a highly localised level [58–62]. However, it remains provisional until additional molecular studies are conducted on ticks derived from small mammals in this part of the world. Prior ectoparasite sampling from rodents trapped in other regions of Saudi Arabia suggest that *Rhipicephalus* OTU 1 might constitute a widespread novel species, and future studies should focus on locating adult specimens to permit a formal description of the taxon.

## Supplementary Information

**Additional file 1: Table S1.** *cox1* interspecific genetic distances among *Rhipicephalus* taxa. **Table S2.** 12S rRNA interspecific genetic distances among *Rhipicephalus* taxa. **Table S3.** 16S rRNA interspecific genetic distances among *Rhipicephalus* taxa. **Table S4.** 16S rRNA interspecific genetic distances among *Haemaphysalis* taxa.

## Data Availability

Sequence data are available from GenBank with identifiers MW742686-MW742711 for *cox1*, MW756110-MW756125 for 12S rRNA, and MW763030-MW763059 for 16S rRNA.

## Abbreviations

ABGD: Automatic barcode gap discovery
OTU: Operational taxonomic unit

## References

[1] Guglielmone A, Robbins RG, Apanaskevich DA, Petney TN, Estrada-Pena A, Horak IG (2010). The Argasidae, Ixodidae and Nuttalliellidae (Acari: Ixodida) of the world: a list of valid species names. Zootaxa.

[2] Murgia MV, Bell-Sakyi L, de la Fuente J, Kurtti TJ, Makepeace BL, Mans B (2019). Meeting the challenge of tick-borne disease control: A proposal for 1000 *Ixodes* genomes. Ticks Tick Borne Dis.

[3] Mans BJ, Featherston J, Kvas M, Pillay KA, de Klerk DG, Pienaar R (2019). Argasid and ixodid systematics: Implications for soft tick evolution and systematics, with a new argasid species list. Ticks Tick Borne Dis.

[4] Kelava S, Mans BJ, Shao R, Moustafa MA, Matsuno K, Takano A (2021). Phylogenies from mitochondrial genomes of 120 species of ticks: Insights into the evolution of the families of ticks and of the genus *Amblyomma*. Ticks Tick Borne Dis..

[5] Kwak ML, Beveridge I, Koehler AV, Malipatil M, Gasser RB, Jabbar A (2017). Phylogenetic analysis of the Australasian paralysis ticks and their relatives (Ixodidae: Ixodes: Sternalixodes). Parasit Vectors.

[6] Black WC, Piesman J (1994). Phylogeny of hard- and soft-tick taxa (Acari: Ixodida) based on mitochondrial 16S rDNA sequences. Proc Natl Acad Sci USA.

[7] Hornok S, Wang Y, Otranto D, Keskin A, Lia RP, Kontschan J (2016). Phylogenetic analysis of *Haemaphysalis erinacei* Pavesi, 1884 (Acari: Ixodidae) from China, Turkey. Italy and Romania Parasit Vectors.

[8] McCann KM, Grant WN, Spratt DM, Hedtke SM (2019). Cryptic species diversity in ticks that transmit disease in Australia. Int J Parasitol Parasites Wildl.

[9] Burger TD, Shao R, Barker SC (2014). Phylogenetic analysis of mitochondrial genome sequences indicates that the cattle tick, *Rhipicephalus* (*Boophilus*) *microplus*, contains a cryptic species. Mol Phylogenet Evol.

[10] Low VL, Prakash BK (2018). First genetic characterization of the brown dog tick *Rhipicephalus sanguineus* sensu lato in Peninsular Malaysia. Exp Appl Acarol.

[11] Roy BC, Estrada-Pena A, Krucken J, Rehman A, Nijhof AM (2018). Morphological and phylogenetic analyses of *Rhipicephalus microplus* ticks from Bangladesh, Pakistan and Myanmar. Ticks Tick Borne Dis.

[12] Low VL, Tay ST, Kho KL, Koh FX, Tan TK, Lim YA (2015). Molecular characterisation of the tick *Rhipicephalus microplus* in Malaysia: new insights into the cryptic diversity and distinct genetic assemblages throughout the world. Parasit Vectors.

[13] Dantas-Torres F, Latrofa MS, Annoscia G, Giannelli A, Parisi A, Otranto D (2013). Morphological and genetic diversity of *Rhipicephalus sanguineus* sensu lato from the New and Old Worlds. Parasit Vectors.

[14] Nava S, Beati L, Venzal JM, Labruna MB, Szabo MPJ, Petney T (2018). *Rhipicephalus sanguineus* (Latreille, 1806): Neotype designation, morphological re-description of all parasitic stages and molecular characterization. Ticks Tick Borne Dis.

[15] Chitimia-Dobler L, Langguth J, Pfeffer M, Kattner S, Kupper T, Friese D (2017). Genetic analysis of *Rhipicephalus sanguineus* sensu lato ticks parasites of dogs in Africa north of the Sahara based on mitochondrial DNA sequences. Vet Parasitol.

[16] Burger TD, Shao R, Barker SC (2013). Phylogenetic analysis of the mitochondrial genomes and nuclear rRNA genes of ticks reveals a deep phylogenetic structure within the genus *Haemaphysalis* and further elucidates the polyphyly of the genus *Amblyomma* with respect to *Amblyomma sphenodonti* and *Amblyomma elaphense*. Ticks Tick Borne Dis.

[17] Puillandre N, Lambert A, Brouillet S, Achaz G (2012). ABGD, Automatic Barcode Gap Discovery for primary species delimitation. Mol Ecol.

[18] Lv J, Wu S, Zhang Y, Zhang T, Feng C, Jia G (2014). Development of a DNA barcoding system for the Ixodida (Acari: Ixodida). Mitochondrial DNA.

[19] Evans ML, Egan S, Irwin PJ, Oskam CL (2019). Automatic Barcode Gap Discovery reveals large COI intraspecific divergence in Australian Ixodidae. Zootaxa.

[20] Kanduma EG, Bishop RP, Githaka NW, Skilton RA, Heyne H, Mwacharo JM (2019). Mitochondrial and nuclear multilocus phylogeny of *Rhipicephalus* ticks from Kenya. Mol Phylogenet Evol.

[21] Hoogstraal H, Kaiser MN. Ticks (Ixodoidea) of Arabia, with special reference to the Yemen. v.39:no.28 (1959). [Chicago]: Chicago Natural History Museum.

[22] Hoogstraal H, Wassef HY, Büttiker W (1981). Ticks (Acarina) of Saudi Arabia, fam Argasidae, Ixodidae. Fauna Saudi Arabia..

[23] Alanazi AD, Alouffi AS, Alshahrani MY, Alyousif MS, Abdullah H, Allam AM (2021). A report on tick burden and molecular detection of tick-borne pathogens in cattle blood samples collected from four regions in Saudi Arabia. Ticks Tick Borne Dis..

[24] Alanazi AD, Nguyen VL, Alyousif MS, Manoj RRS, Alouffi AS, Donato R (2020). Ticks and associated pathogens in camels (*Camelus dromedarius*) from Riyadh Province, Saudi Arabia. Parasit Vectors.

[25] Chandra S, Smith K, Alanazi AD, Alyousif MS, Emery D, Slapeta J (2019). *Rhipicephalus sanguineus* sensu lato from dogs and dromedary camels in Riyadh, Saudi Arabia: low prevalence of vector-borne pathogens in dogs detected using multiplexed tandem PCR panel. Folia Parasitol (Praha).

[26] Alanazi AD, Al-Mohammed HI, Alyousif MS, Said AE, Salim B, Abdel-Shafy S (2019). Species diversity and seasonal distribution of hard ticks (Acari: Ixodidae) infesting mammalian hosts in various districts of Riyadh Province, Saudi Arabia. J Med Entomol.

[27] Harrison A, Robb GN, Alagaili AN, Hastriter MW, Apanaskevich DA, Ueckermann EA (2015). Ectoparasite fauna of rodents collected from two wildlife research centres in Saudi Arabia with discussion on the implications for disease transmission. Acta Trop.

[28] Asiry KA, Fetoh BS (2014). Occurrence of ectoparasitic arthropods associated with rodents in Hail region northern Saudi Arabia. Environ Sci Pollut Res Int.

[29] Stekolnikov AA, Al-Ghamdi SQ, Alagaili AN, Makepeace BL (2019). First data on chigger mites (Acariformes: Trombiculidae) of Saudi Arabia, with a description of four new species. Syst Appl Acarol.

[30] Harrison DL, Bates PJJ (1991). The mammals of Arabia.

[31] Kocher TD, Thomas WK, Meyer A, Edwards SV, Paabo S, Villablanca FX (1989). Dynamics of mitochondrial DNA evolution in animals: amplification and sequencing with conserved primers. Proc Natl Acad Sci USA.

[32] Hoogstraal H (1958). Notes on African Haemaphysalis ticks. IV. Description of Egyptian populations of the yellow dog-tick, *H. leachii leachii* (Audouin, 1827) (Ixodoidea, Ixodidae). J Parasitol.

[33] Morel PC, Mouchet J, Rodhain F (1976). Description of *Rhipicephalus camicasi *n. sp. (Acaridae, Ixodida) of subdesert steppes of the plain of Afar. Rev Elev Med Vet Pays Trop.

[34] Pegram RG, Walker JB, Clifford CM, Keirans JE (1987). Comparison of populations of the *Rhipicephalus simus* group: *R. simus*, *R. praetextatus*, and *R. muhsamae* (Acari: Ixodidae). J Med Entomol.

[35] Pegram RG, Clifford CM, Walker JB, Keirans JE (1987). Clarification of the *Rhipicephalus sanguineus* group (Acari, Ixodoidea, Ixodidae). 1. *Rhipicephalus sulcatus* Neumann, 1908 and *Rhipicephalus turanicus* Pomerantsev, 1936. Syst Parasitol.

[36] Pegram RG, Keirans JE, Clifford CM, Walker JB (1987). Clarification of the *Rhipicephalus sanguineus* group (Acari, Ixodoidea, Ixodidae). 2. *Rhipicephalus sanguineus* (Latreille, 1806) and related species. Syst Parasitol.

[37] Apanaskevich DA, Horak IG, Camicas JL (2007). Redescription of *Haemaphysalis (Rhipistoma) elliptica* (Koch, 1844), an old taxon of the *Haemaphysalis (Rhipistoma) leachi* group from East and southern Africa, and of *Haemaphysalis (Rhipistoma) leachi* (Audouin, 1826) (Ixodida, Ixodidae). Onderstepoort J Vet Res.

[38] Estrada-Pena A, Mihalca AD, Petney TN (2017). Ticks of Europe and North Africa: a guide to species identification.

[39] Beati L, Keirans JE (2001). Analysis of the systematic relationships among ticks of the genera *Rhipicephalus* and *Boophilus* (Acari: Ixodidae) based on mitochondrial 12S ribosomal DNA gene sequences and morphological characters. J Parasitol.

[40] Altschul SF, Gish W, Miller W, Myers EW, Lipman DJ (1990). Basic local alignment search tool. J Mol Biol.

[41] Thompson JD, Gibson TJ, Plewniak F, Jeanmougin F, Higgins DG (1997). The CLUSTAL_X windows interface: flexible strategies for multiple sequence alignment aided by quality analysis tools. Nucleic Acids Res.

[42] Hall TA (1999). BioEdit: a user-friendly biological sequence alignment editor and analysis program for Windows 95/98/NT. Nucleic Acids Symp Ser.

[43] Kumar S, Stecher G, Li M, Knyaz C, Tamura K (2018). MEGA X: Molecular Evolutionary Genetics Analysis across computing platforms. Mol Biol Evol.

[44] Hillis DM, Bull JJ (1993). An empirical test of bootstrapping as a method for assessing confidence in phylogenetic analysis. Syst Biol.

[45] Swofford D. PAUP*. Phylogenetic Analysis Using Parsimony (*and Other Methods). Version 4.0b10. vol. Version 4.0; 2002.

[46] Slapeta J, Chandra S, Halliday B (2021). The, "tropical lineage" of the brown dog tick* Rhipicephalus sanguineus* sensu lato identified as* Rhipicephalus linnae*i (Audouin, 1826). Int J Parasitol.

[47] Hornok S, Sandor AD, Tomanovic S, Beck R, D'Amico G, Kontschan J (2017). East and west separation of *Rhipicephalus sanguineus* mitochondrial lineages in the Mediterranean Basin. Parasit Vectors.

[48] Zemtsova GE, Apanaskevich DA, Reeves WK, Hahn M, Snellgrove A, Levin ML (2016). Phylogeography of *Rhipicephalus sanguineus* sensu lato and its relationships with climatic factors. Exp Appl Acarol.

[49] Bakkes DK, Chitimia-Dobler L, Matloa D, Oosthuysen M, Mumcuoglu KY, Mans BJ (2020). Integrative taxonomy and species delimitation of *Rhipicephalus turanicus* (Acari: Ixodida: Ixodidae). Int J Parasitol.

[50] El-Azazy OM, Scrimgeour EM (1997). Crimean-Congo haemorrhagic fever virus infection in the western province of Saudi Arabia. Trans R Soc Trop Med Hyg.

[51] Sanborn CC, Hoogstraal H (1953). Some mammals of Yemen and their ectoparasites.

[52] Islam MM, Farag E, Eltom K, Hassan MM, Bansal D, Schaffner F (2021). Rodent ectoparasites in the Middle East: A systematic review and meta-analysis. Pathogens.

[53] Thompson AT, Dominguez K, Cleveland CA, Dergousoff SJ, Doi K, Falco RC (2020). Molecular characterization of *Haemaphysalis* species and a molecular genetic key for the identification of *Haemaphysalis* of North America. Front Vet Sci.

[54] Tomlinson JA, Horak IG, Apanaskevich DA (2018). Identity of *Haemaphysalis* (*Rhipistoma*) *muhsamae* Santos Dias, 1954 (Acari: Ixodidae) and *H*. (*R*.) *subterra* Hoogstraal, El Kammah & Camicas, 1992, parasites of carnivores and rodents in eastern and southern Africa. Syst Parasitol.

[55] Hekimoglu O, Saglam IK, Ozer N, Estrada-Pena A (2016). New molecular data shed light on the global phylogeny and species limits of the *Rhipicephalus sanguineus* complex. Ticks Tick Borne Dis.

[56] Scott JD, Foley JE, Young MR, Durden LA (2017). First report of a blacklegged tick, *Ixodes scapularis* Say (Acari: Ixodidae), parasitizing a raptor in Canada. Syst Appl Acarol.

[57] Leo SS, Pybus MJ, Sperling FA (2010). Deep mitochondrial DNA lineage divergences within Alberta populations of *Dermacentor albipictus* (Acari: Ixodidae) do not indicate distinct species. J Med Entomol.

[58] Al-Khafaji AM, Clegg SR, Pinder AC, Luu L, Hansford KM, Seelig F (2019). Multi-locus sequence typing of *Ixodes ricinus* and its symbiont *Candidatus* Midichloria mitochondrii across Europe reveals evidence of local co-cladogenesis in Scotland. Ticks Tick Borne Dis.

[59] Jia N, Wang J, Shi W, Du L, Sun Y, Zhan W (2020). Large-scale comparative analyses of tick genomes elucidate their genetic diversity and vector capacities. Cell.

[60] Poli P, Lenoir J, Plantard O, Ehrmann S, Roed KH, Leinaas HP (2020). Strong genetic structure among populations of the tick *Ixodes ricinus* across its range. Ticks Tick Borne Dis..

[61] Xu G, Wielstra B, Rich SM (2020). Northern and southern blacklegged (deer) ticks are genetically distinct with different histories and Lyme spirochete infection rates. Sci Rep.

[62] Lado P, Smith ML, Carstens BC, Klompen H (2020). Population genetic structure and demographic history of the lone star tick, *Amblyomma americanum* (Ixodida: Ixodidae): New evidence supporting old records. Mol Ecol.

[63] Underwood W, Anthony R, Cartner S, Corey D, Grandin T, Greenacre CB (2013). AVMA guidelines for the euthanasia of animals.

[64] Charbonneau R, Niel L, Olfert E, von Keyserlingk M, Griffin C (2010). CCAC guidelines on: Euthanasia of animals used in science.

