# Supplementary material for: Automatic barcode gap discovery reveals diverse clades of Rhipicephalus spp. and Haemaphysalis spp. ticks from small mammals in 'Asir, Saudi Arabia
Source: Parasit Vectors. 2021 Oct 19;14:541. doi: 10.1186/s13071-021-05049-x (PMC8527726; doi:10.1186/s13071-021-05049-x)
Supplement: Supplementary file 1 — Additional file 1: Table S1. cox1 interspecific genetic distances among Rhipicephalus taxa. Table S2. 12S rRNA interspecific genetic distances among Rhipicephalus taxa. Table S3. 16S rRNA interspecific genetic distances among Rhipicephalus taxa. Table S4. 16S rRNA interspecific genetic distances among Haemaphysalis taxa. [file 13071_2021_5049_MOESM1_ESM.pdf]

Table S1 *coi* interspecific genetic distances among *Rhipicephalus* taxa

		1	2	3	4	5	6	7	8	9	10	11	12	13	14	15
**1.**	***R.* sp. (OTU 1, this study)**	-														
**2.**	***R. leporis***	2.0-2.78 (2.2)	-													
**3.**	***R. linnaei***	2.8-3.2 (2.9)	1.6-2.4 (2.0)	-												
**4.**	***R. guilhoni***	3.9-5.1 (4.6)	4.3-5.5 (4.9)	5.1-6.3 (5.5)	-											
**5.**	***R.* cf. *camicasi***	3.9-5.5 (4.8)	5.1-5.9 (5.5)	5.1-6.3 (5.6)	4.7-5.9 (5.3)	-										
**6.**	***R. sanguineus* s.l., south-eastern European lineage**	8.3-9.0 (8.7)	7.1-7.9 (7.5)	8.3-9.1 (8.5)	7.5-7.9 (7.7)	7.1-7.5 (7.2)	-									
**7.**	***R.* sp. Nigeria (OTU 12)**	9.5-10.2 (9.9)	10.6-11.0 (10.9)	10.6-11.0 (10.7)	10.6-11.0 (10.8)	9.1-9.5 (9.3)	7.9 (7.9)	-								
**8.**	***R. turanicus***	9.8-11.4 (10.7)	10.2-11.4 (10.8)	9.5-11.4 (10.1)	9.8-11.0 (10.4)	9.1-10.6 (9.8)	7.5-8.3 (7.9)	8.3-9.1 (8.7)	-							
**9.**	***R. sanguineus* s.s.**	9.8-19.6 (10.3)	9.1 (9.1)	9.5-10.2 (9.5)	10.2-11.4 (10.8)	9.5-10.2 (9.7)	7.9 (7.9)	8.7-9.1 (8.81)	9.1-9.8 (9.5)	-						
**10.**	***R. pusillus***	13.0-13.8 (13.4)	12.2-12.6 (12.5)	13.0-13.4 (13.3)	11.0-11.4 (11.2)	12.6-13.40 (13.0)	10.2 (10.2)	11.8 (11.8)	10.6-11.0 (10.8)	11.0-11.4 (11.2)	-					
**11.**	***R. rossicus***	11.4-12.6 (12.0)	11.4-12.2 (11.8)	11.4-12.2 (11.7)	10.2-10.6 (10.4)	9.5-10.6 (10.1)	7.9-8.3 (8.0)	6.7-7.9 (7.4)	7.9-9.0 (8.4)	9.8-11.0 (10.5)	9.8-10.6 (10.2)	-				
**12.**	***R.* sp. Kenya (OTU 14)**	14.2-15.8 (15.0)	14.6-16.1 (15.4)	15.0-15.8 (15.4)	13.0-14.2 (13.6)	13.4-14.6 (13.9)	11.8-12.2 (11.9)	12.6-13.0 (12.9)	11.8-13.40 (12.6)	13.0-14.6 (13.7)	13.8-14.2 (14.0)	11.4-12.2 (11.7)	-			
**13.**	***R. muhsamae***	16.1-16.9 (16.6)	15.8-16.5 (16.1)	16.9-17.3 (17.0)	14.2-14.6 (14.4)	14.0-15.8 (15.4)	11.4 (11.4)	13.7 (13.8)	14.6-15.4 (15.0)	15.0-15.8 (15.3)	13.4 (13.4)	12.6-13.4 (13.0)	5.5-6.7 (6.3)	-		
**14.**	***R. praetextatus***	14.6-15.4 (15.0)	15.0-15.8 (15.4)	14.6-15.4 (14.8)	14.2-14.6 (14.4)	14.6-15.0 (14.8)	12.6 (12.6)	11.4-12.2 (11.7)	12.2-13.0 (12.6)	12.6-14.6 (13.2)	14.2 (14.2)	11.4-12.2 (11.8)	8.3-9.5 (8.8)	8.7 (8.7)	-	
**15.**	***R. simus***	14.6-16.1 (15.4)	15.0-16.1 (15.6)	15.0-16.1 (15.5)	14.6-15.4 (14.9)	14.6-15.4 (15.1)	13.0-13.4 (13.1)	12.2-12.6 (12.5)	14.2-15.8 (15.0)	13.8-14.6 (14.3)	14.6-15.0 (14.8)	11.8-13.0 (12.5)	8.7-9.5 (9.0)	8.3-8.7 (8.4)	2.8-3.9 (3.3)	-

Table S2 12S rRNA interspecific genetic distances among *Rhipicephalus* taxa

		1	2	3	4	5	6	7	8	9	10	11	12
**1.**	***R.* sp. (OTU 1, this study)**	-											
**2.**	***R. leporis***	1.8 (1.8)	-										
**3.**	***R. linnaei***	1.4-2.3 (1.8)	0.9-1.8 (1.4)	-									
**4.**	***R. guilhoni***	2.3 (2.3)	1.8 (1.8)	0.9-1.8 (1.4)	-								
**5.**	***R.* cf. *camicasi***	2.8-4.2 (3.3)	3.2-4.6 (3.6)	3.7-6.0 (4.7)	3.7-5.5 (4.4)	-							
**6.**	***R. sanguineus* s.l., south-eastern European lineage**	5.5-6.5 (6.0)	6.0-6.9 (6.5)	6.5-8.3 (7.3)	5.5-6.5 (6.0)	2.7-6.0 (4.3)	-						
**7.**	***R.* sp. Nigeria (OTU 7)**	8.8 (8.8)	8.3 (8.3)	8.8-9.7 (9.3)	8.3 (8.3)	6.5-7.4 (7.2)	6.0-7.0 (6.6)	-					
**8.**	***R. turanicus***	6.5-6.9 (6.7)	6.0-6.5 (6.2)	6.5-7.4 (7.0)	5.5-6.0 (5.8)	4.2-6.0 (5.3)	5.1-6.5 (5.8)	5.6-6.0 (5.8)	-				
**9.**	***R. sanguineus s.s.***	8.0-8.9 (8.3)	8.9-9.8 (9.2)	8.4-10.2 (9.1)	7.5-8.4 (7.8)	6.1-8.9 (7.7)	5.6-8.0 (7.0)	7.5-8.9 (8.0)	4.7-6.6 (5.6)	-			
**10.**	***R. pusillus***	7.9-8.3 (8.2)	8.8-9.3 (9.1)	8.4-9.3 (9.0)	8.4-8.8 (8.7)	7.0-8.3 (7.7)	7.4-8.8 (8.2)	8.8-9.3 (9.1)	6.5-7.4 (7.0)	5.1-6.5 (5.8)	-		
**11.**	***R. rossicus***	8.3 (8.3)	8.8 (8.8)	9.2-9.7 (9.5)	8.3 (8.3)	6.5-7.4 (7.1)	6.0-6.9 (6.5)	8.3 (8.3)	6.5-6.9 (6.7)	6.6-8.0 (7.3)	5.1-5.5 (5.4)	-	
**12.**	***R. simus* group***	10.6-11.6 (11.1)	12.0-13.0 (12.5)	11.2-13.4 (12.6)	11.1-12.0 (11.5)	9.7-11.6 (10.7)	7.4-9.8 (8.6)	7.5-10.2 (9.0)	8.3-9.3 (8.9)	5.2-7.5 (6.5)	5.5-7.4 (6.5)	6.9-7.9 (7.4)	-

***Includes R29_larvae pool.**

Table S3. 16S rRNA interspecific genetic distances among *Rhipicephalus* taxa

		1	2	3	4	5	6	7	8	9	10	11	12
**1.**	***R.* sp. (OTU 1, this study)**	-											
**2.**	***R. linnaei***	4.4-4.8<br>(4.5)	-										
**3.**	***R. guilhoni***	3.5-4.8<br>(4.1)	1.8-2.2<br>(2.0)	-									
**4.**	***R.* cf. *camicasi***	6.1-7.9<br>(6.9)	5.7-6.6<br>(6.2)	4.8-5.7<br>(5.2)	-								
**5.**	***R. sanguineus* s.l., south-eastern European lineage**	6.1-7.0<br>(6.4)	4.4-4.8<br>(4.5)	3.5-4.4<br>(3.9)	4.4-5.7<br>(5.1)	-							
**6.**	***R.* sp. Nigeria (OTU 6)**	9.7-11.0<br>(10.3)	9.3-10.1<br>(9.7)	8.4-9.7<br>(9.0)	8.0-9.3<br>(8.8)	7.5-8.8<br>(8.1)	-						
**7.**	***R. turanicus***	8.7-10.1<br>(9.3)	7.4-9.2<br>(8.0)	7.0-8.8<br>(7.7)	6.1-10.1<br>(7.7)	4.4-7.0<br>(5.3)	8.4-9.2<br>(8.8)	-					
**8.**	***R. sanguineus s.s.***	9.7-11.7<br>(10.3)	8.8-10.7<br>(9.3)	8.4-10.8<br>(9.1)	6.2-9.4<br>(7.5)	6.2-8.5<br>(6.8)	10.2-12.6<br>(10.9)	6.6-10.8<br>(8.2)	-				
**9.**	***R. pusillus***	9.7-10.3<br>(10.0)	10.1-11.0<br>(10.4)	9.7-11.0<br>(10.3)	10.6-12.3<br>(11.4)	8.9-9.7<br>(9.2)	12.9-14.0<br>(13.5)	10.6-12.5<br>(11.2)	10.7-12.6<br>(11.2)	-			
**10.**	***R. rossicus***	10.5-12.2<br>(11.4)	10.9-11.3<br>(11.1)	9.2-11.4<br>(10.3)	11.0-12.7<br>(11.8)	8.3-8.8<br>(8.5)	13.2-14.5<br>(13.8)	11.4-12.7<br>(11.9)	8.8-11.1<br>(9.7)	9.7-10.9<br>(10.2)	-		
**11.**	***R. muhsamae***	11.3-13.3<br>(12.3)	10.4-11.5<br>(11.1)	9.5-11.5<br>(10.6)	9.9-12.9<br>(11.4)	9.9-11.5<br>(10.8)	14.0-15.9<br>(15.0)	10.3-15.1<br>(12.0)	10.8-14.7<br>(12.1)	10.3-11.4<br>(11.9)	12.6-15.0<br>(13.7)	-	
**12.**	***R. simus***	11.4-12.9<br>(11.9)	9.2-10.7<br>(9.6)	8.8-10.7<br>(9.3)	9.3-12.0<br>(10.5)	8.8-10.7<br>(9.3)	10.6-13.7<br>(11.8)	9.3-13.4<br>(10.8)	10.6-13.4<br>(11.4)	11.9-13.0<br>(12.3)	12.4-14.2<br>(13.2)	6.6-9.8<br>(7.6)	-

Table S4. 16S rRNA interspecific genetic distances among *Haemaphysalis* taxa

		1	2	3	4	5	6	7	8	9	10	11	12	13	14	15	16
**1.**	***H.* sp. (OTU 1, this study)**	-															
**2.**	***H. spinulosa***	7.1-7.7 (7.38)	-														
**3.**	***H. parva***	9.3-9.9 (9.6)	8.9-9.0 (9.0)	-													
**4.**	***H.* sp. (OTU 4, this study)**	7.8-8.6 (8.2)	9.6-9.8 (9.7)	8.6-8.7 (8.6)	-												
**5.**	***H. muhsamae***	9.6-10.2 (9.9)	9.9 (9.9)	9.6-9.6 (9.6)	6.5-7.1 (6.8)	-											
**6.**	***H. elliptica***	10.5-11.2 (10.9)	11.12 (11.2)	9.9 (9.9)	8.1-8.3 (8.2)	6.5 (6.5)	-										
**7.**	***H. kopetdaghica***	10.0-10.3 (10.1)	12.8 (12.8)	14.0 (14.0)	12.0-12.1 (12.0)	14.1 (14.1)	13.2 (13.2)	-									
**8.**	***H. hystricis***	11.3-11.6 (11.6)	11.0 (11.0)	9.7-10.3 (10.0)	10.4-11.3 (10.9)	11.3-12.0 (11.7)	12.0-12.7 (12.3)	11.3-11.6 (11.5)	-								
**9.**	***H. sulcata***	12.8-13.4 (13.1)	10.3 (10.3)	10.9-11.5 (11.2)	10.4-11.8 (11.1)	10.3-10.6 (10.5)	12.6-12.9 (12.7)	14.6-14.6 (14.6)	11.4-12.00 (11.7)	-							
**10.**	***H. longicornis***	14.0-14.9 (14.3)	14.9-15.7 (15.2)	12.7-13.3 (13.0)	11.0-12.0 (11.5)	11.6-12.1 (11.8)	12.2-12.8 (12.5)	15.4-15.6 (15.5)	10.0-11.3 (10.7)	10.0-10.6 (10.3)	-						
**11.**	***H. e. turanica***	13.1-14.6 (13.8)	11.2-12.1 (11.6)	11.1-12.0 (11.5)	8.7-9.8 (9.3)	11.5-12.1 (11.8)	10.6-11.5 (11.0)	13.5-14.4 (14.0)	11.7-13.0 (12.3)	10.3-11.5 (10.9)	11.96-13.3 (12.6)	-					
**12.**	***H. e. taurica***	13.4-13.7 (13.7)	11.9-12.5 (12.2)	11.1-13.5 (11.3)	10.3-11.7 (10.9)	11.5-12.1 (11.8)	11.2-11.8 (11.5)	13.6-13.9 (13.7)	11.1-11.4 (11.2)	9.1-9.7 (9.5)	10.3-11.5 (10.9)	4.9-5.9 (5.4)	-				
**13.**	***H. e. erinacei***	13.4-13.7 (13.5)	11.8-12.1 (12.0)	11.4-11.7 (11.6)	10.6-11.4 (11.0)	12.4-12.7 (12.6)	12.2-12.4 (12.3)	12.9-13.2 (13.1)	10.7-11.0 (10.9)	9.7 (9.7)	11.1-11.2 (11.2)	5.2-6.4 (5.8)	1.9-2.2 (2.0)	-			
**14.**	***H. punctata***	15.0-15.3 (15.1)	12.8 (12.8)	12.8-12.8 (12.8)	13.0-13.2 (13.1)	15.3 (15.3)	15.1 (15.1)	13.9 (13.9)	13.9 (13.9)	15.1-15.4 (15.3)	15.8-15.9 (15.9)	13.9-15.8 (15.4)	14.7-15.6 (15.1)	14.6-14.7 (14.6)	-		
**15.**	***H. flava***	13.4-14.0 (13.6)	14.0 (14.0)	11.5-11.9 (11.7)	10.9-11.3 (11.1)	12.8-13.2 (13.0)	12.9-13.2 (13.1)	14.2-14.9 (14.6)	15.7-16.4 (16.1)	15.1-15.4 (15.2)	14.9-15.3 (15.1)	14.1-15.0 (14.5)	14.4-15.4 (14.9)	14.4-14.4 (14.4)	10.9-11.3 (11.1)	-	
**16.**	***H. conconna***	14.1-14.6 (14.2)	14.0-14.3 (14.1)	13.6-13.9 (13.8)	13.0-13.3 (13.1)	14.3-14.4 (14.4)	12.5-12.8 (12.7)	14.8 (14.8)	16.1-16.4 (16.2)	16.0-16.7 (16.3)	16.2-16.8 (16.5)	15.9-17.0 (16.4)	16.2-17.1 (16.8)	16.5-17.1 (16.8)	10.3-10.9 (10.6)	8.8-9.7 (9.2)	-
